# Is Meat of Breeder Turkeys so Different from That of Standard Turkeys?

**DOI:** 10.3390/foods8010008

**Published:** 2018-12-24

**Authors:** Pascal Chartrin, Thierry Bordeau, Estelle Godet, Karine Méteau, Jean-Christian Gicquel, Estelle Drosnet, Sylvain Brière, Marie Bourin, Elisabeth Baéza

**Affiliations:** 1 Biologie des Oiseaux et Aviculture (BOA), Institut National de la Recherche Agronomique (INRA), Université de Tours, 37380 Nouzilly, France; pascal.chartrin@inra.fr (P.C.); thierry.bordeau@inra.fr (T.B.); estelle.godet@inra.fr (E.G.); 2 Elevage, Alimentation et Santé des Monogastriques (EASM), INRA, Le Magneraud, Saint-Pierre d’Amilly, BP 52, 17700 Surgères, France; karine.meteau@inra.fr; 3 Société de Transformation des Volailles de l’Ouest (STVO), ZI les Riantières, BP 22, 44540 Saint-Mars la Jaille, France; Jean-Christian.GICQUEL@hendrix-genetics.com (J.-C.G.); estelle.DROSNET@hendrix-genetics.com (E.D.); 4 Hendrix Genetics Turkeys France, La Bohardière, BP 1, St Laurent de la Plaine, 49290 Mauges Sur Loire, France; sylvain.briere@hendrix-genetics.com; 5 Institut Technique de l’Aviculture (ITAVI), BOA, INRA, Université de Tours, 37380 Nouzilly, France; bourin@itavi.asso.fr

**Keywords:** male and female turkeys, breeders, broilers, carcass, meat, nutritional, sensorial and technological quality

## Abstract

The technological, nutritional, and sensorial quality of breasts and thighs with drumsticks of turkey male and female breeders was characterized by comparison with breasts and thighs with drumsticks of growing male and female turkeys from the Grademaker line (hybrid turkeys, *n* = 20 birds per sex and per physiological stage). The breeder turkeys were slaughtered at 397 and 410 days of age and 10.42 and 32.67 kg of body weight for the females and males, respectively. The standard turkeys were slaughtered at 75 and 103 days of age and 5.89 and 13.48 kg of body weight for the females and males, respectively. The differences observed between males and females on one hand and between standard and breeder turkeys on the other hand were mainly induced by differences in slaughter ages and sexual dimorphism on body weight. The meat of female breeders had characteristics close to those of female and male standard turkeys, whereas the meat of male breeders was clearly distinguishable, particularly by displaying lower tenderness and water holding capacity.

## 1. Introduction

In France, the production of turkey meat reached 350,000 tec (tonnes equivalent carcasses) in 2015 [1]. The consumption of turkey meat was 4.6 kg per year and per capita in 2015 [2]. This production results essentially from standard turkeys. Females and males are slaughtered at 12 and 16 weeks of age, respectively, and 6–7 kg and 14–15 kg of body weight, respectively. The main part of the turkey production is cut, but there is a production of light turkeys sold under whole carcasses around Christmas and New Year holidays. Finally, when the period of reproduction of turkeys is finished, animals are slaughtered under industrial conditions and their meat is mainly valued under processed products. The French production of breeders was estimated to 8797 and 9174 tec in 2015 and 2016, respectively (Gicquel, personal communication). In the EU, production was estimated to 36,224 and 37,776 tec in 2015 and 2016, respectively (Gicquel, personal communication). There are many studies on the meat quality of standard turkeys. In other countries, standard turkeys are slaughtered at older ages (18 to 22 weeks for males, and 14 to 16 weeks for females) depending on the line (BUT Big 6, Hybrid Converter). The body weight at slaughter ranges between 16 and 22 kg for males, and between 9 and 11 kg for females [3,4,5,6,7,8,9,10,11,12,13,14,15]. The breast yield can vary between 22 and 30% and the yield of thighs with drumsticks can vary between 19 and 28% depending on the line, the slaughter age, and the sex. The range of ultimate pH comprises between 5.55 and 6.20 in breast muscle and between 5.75 and 6.30 in thigh muscle [3,4,5,9,16,17,18,19,20,21,22,23,24,25,26,27,28]. The juice loss can vary from 1 to 6% depending on the duration of cold storage and the cooking loss from 4% to 29% depending on the study and probably the cooking conditions and duration [7,8,9,10,12,14,15,19,23,25,28]. The shear force value of breast muscle ranges between 7 and 46 N depending on the study (cooked or raw meat). Some studies also reported the chemical composition of standard turkey meat. The water, protein, lipid, and ash contents of breast muscle vary between 72 and 75%, 21 and 28%, 0.4 and 4.0%, and 1.0 and 1.3%, respectively [3,5,7,9,10,12,14,15,16,20,24,26,27,28]. On the other hand, to our knowledge, no study has been published on the meat characteristics of male and female breeder turkeys. The aim of the present study was to evaluate the technological, nutritional, and sensorial quality of meat from breeder turkeys in comparison with that of standard turkeys.

## 2. Materials and Methods

### 2.1. Experimental Design

In order to realize this characterization, carcasses of male and female breeder turkeys from the Grademaker line (Hybrid Turkeys) were compared with carcasses of male and female standard turkeys from the same line (*n* = 20 per sex and per physiological stage). Hendrix Genetics Turkeys Company (Saint-Laurent de la Plaine, France) provided the animals reared, according the breeder recommendations. The turkeys were slaughtered according to standard procedures, which include immobilization by electrical stunning, followed by exsanguination, defeathering, and evisceration (=D0). STVO (Société de Transformation des Volailles de l’Ouest) Company (Saint-Mars la Jaille, France) cut the carcasses 24 h after slaughter and cold storage at 4 °C (=D1) in order to determine the meat yields. The thighs with drumsticks and breast muscles were then transported and stored under refrigerated conditions to the Research Unit BOA (Biologie des Oiseaux et Aviculture, INRA Nouzilly, France). From every left fillet, 4 cutlets of 200 g and 2 cm in thickness were individually cut and packed in a bag (sealed air cryovac, 60 µm) under vacuum (multivac P300, Cenpac, Chambray les Tours, France) and identified. The left thighs and drumsticks, with bones and skin, were individually packed in a bag, under vacuum, and identified. These samples were transported under refrigerated conditions to the experimental unit EASM (Elevage, Alimentation et Santé des Monogastriques, INRA Magneraud, Surgères, France) and stored at −20 °C for further sensorial analysis.

### 2.2. Analysis of Technological Quality of Meat

The right fillets and thighs with drumsticks were used to realize various measures and to take several samples on day 2. The ultimate pH (pHu) was determined by direct insertion of an electrode (pH meter Model 506, Crison Instruments, Barcelona, Spain) into the Pectoralis major (PM) and Iliotibialis superficialis (IT) muscles. The color was measured on the same muscles by using a Miniscan Spectrocolorimeter (Hunterlab, Reston, VA, USA) with the CIELAB thrichromatic system as lightness (L*), redness (a*), and yellowness (b*) values. The water holding capacity was estimated by measuring drip loss of a raw cutlet (around 150 g) placed in a plastic bag, hung from a hook, and stored at 4 °C for 6 days. The drip loss was expressed as the percentage of the initial cutlet weight. The cooking loss was measured on a thick cutlet (around 150–200 g) and packed in a bag under vacuum on day 3. The cutlets were cooked in a water bath at 85 °C for 16 min. They were then cooled during 15 min in crushed ice. The cooking loss was expressed as the percentage of the initial cutlet weight. The texture measurement was then realized on these cooked cutlets. The average Warner-Bratzler shear force value was determined on 3 strips (1 cm × 1 cm × 3 cm) for each cooked cutlet [29]. A piece of breast muscle (around 100 g) was processed into cured–cooked meat on day 4 in order to determine the technological yield [30]. 

### 2.3. Analysis of Nutritional Quality of Meat

In breast and thigh muscles, the content in haeminic pigments [31], protein content (Kjeldhal method), moisture content by differential weighing of 5 g of sample placed in steam room at 105 °C during 24 h, and lipid content were determined [32]. Lipids were then methylated [33] and the fatty acid composition was determined by gas chromatography (Perkin Elmer, Saint-Quentin en Yvelines, France) [34]. The classes of lipids were determined using Iatroscan (Iatron, Tokyo, Japan) based on thin-layer chromatography and a flame-ionisation-detector system (TLC–FID) [35]. The lipid peroxidation was evaluated [36] to determine the TBARS (Thio-Barbituric Acid Reactive Substances) value. The protein oxidation was evaluated by measuring the thiol and carbonyl content [37,38].

### 2.4. Analysis of Sensorial Quality of Meat

The sessions of sensory analysis were realized in the laboratory INRA of Magneraud (Surgères, France) in conditions corresponding to the standard [39]. The thighs with drumsticks and cutlets were defrosted before cooking for 24 to 48 h depending on the weight. The thighs with drumsticks were cooked in the oven (25 min at 250 °C, then maintained at 100 °C under wet heat in order to reach a core temperature of 80 °C). The cutlets placed between two aluminum foils were cooked in a steakhouse (5 min in 250 °C to reach a core temperature of 80 °C). Twelve panelists were first trained during three sessions and tasting one turkey per group and per session. Then, the panelists tasted the four groups in every session. Ten sessions were realized, the panelists testing one turkey per group and per session. For every criterion, the notation was made on a continuous scale limited from 0 to 10. Assessment criteria for the tasting of thighs were: Color, tenderness, juiciness, stringiness, compactness, oily sensation, global and rancid flavors, and global appreciation. Assessment criteria for the tasting of cutlets were: Color, tenderness, juiciness, stringiness, sticky, global and rancid flavors, and global appreciation. 

### 2.5. Histological Analysis of PM Muscle

Samples of PM muscle were taken along a line parallel to the fiber axis on day 2 and frozen in isopentane cooled with liquid nitrogen and stored at −80 °C until histological analysis was performed [40]. Serial cross sections, 10 µm thick, were realized with a cryotome. The labeling of type VI collagen of chicken (Developmental Studies Hybridoma Bank, University of Iowa, Iowa City, IA, USA) was realized thanks to the kit Vectastain ABC elite (Laboratoires Eurobio/Abcys, Les Ulis, France) (Mouse IgG, Vector laboratories PK 6102 distributed by Eurobio Ingen (Les Ulis, France)) and revealed with DAB (Sigma, Saint-Quentin Fallavier, France). The cross-sectional area (CSA) of muscle fibers and the relative area occupied by collagen was determined using a computerized image analysis system (Visilog software, Noesis, Crolles, France) on 13 samples per group. 

### 2.6. Statistical Analysis

Data were tested with a variance analysis using Statview software (SAS Institute Inc., Cary, NC, USA). The effects of sex, physiological stage, and their interaction were analyzed by comparing means with a *t*-test and a *p* value < 0.05. Pearson correlations were calculated between different measured parameters and considered significant with *p* < 0.05.

## 3. Results

### 3.1. Meat Yields (Table 1)

The breeders were slaughtered at older ages and they had a higher weight and higher yield of carcass and breast than standard birds. For the yield of thigh with drumstick, it was the opposite observation, particularly for the females. The most important observed differences concerned males compared to females and this whatever the physiological stage (standard or breeder). Indeed, the body weight of male breeders was 3.1 times higher than that of female breeders and the body weight of standard males was 2.3 times higher to that of standard females. Such differences were also reflected on the weight of carcass, breasts, and thighs with drumsticks. The carcass yield of male breeders was higher than that of female breeders, for which the ovaries removed during evisceration weighted approximately 270 g. The carcass yield of standard males was also higher than that of standard females. The yields of breasts and thighs with drumsticks of male breeders were higher than those of female breeders. It was the same for the standard turkeys.

### 3.2. Technological Quality of Meat (Table 2)

The average pHu measured in the PM muscle was 5.70 whatever the sex or the physiological stage. The standard females had a higher pHu in PM muscle than that of standard males. The breeder turkeys had a lower pHu in IT muscle than that of standard turkeys. For the two physiological stages, the males had a lower pHu in IT than that of the females. 

The standard males had darker PM muscle than the other groups and female breeders had darker IT muscle than the other groups. The standard turkeys had PM and IT muscles less red than those of the breeder turkeys, and the females had PM and IT muscles less red than those of the males. The male breeders had PM and IT muscles less yellow than those of the other groups. On the other hand, the females had IT muscles more yellow than the males. 

The male breeders had PM muscles harder than those of the other groups. Globally, the drip loss of PM muscle after a storage at 4 °C was low. It was higher for the males compared to the females. The difference was mostly important for the breeder turkeys (×1.7). The cooking loss of PM muscle was higher for males compared to females and for breeder turkeys, particularly the males, compared to standard turkeys. The PM muscle of males had lower technological yield than that of females and the PM muscle of breeder turkeys, particularly the males, had lower technological yield than that of standard turkeys. Indeed, the female breeders had the highest technological yield. This confirmed the observations on the previous parameters concerning the water holding capacity of PM muscle. The coefficients of correlation between drip loss after a storage at 4 °C and cooking loss or technological yield were 0.46 and 0.63, respectively (*p* < 0.05). The coefficient of correlation between cooking loss and technological yield was 0.67 (*p* < 0.05). 

The protein oxidation in meat was low. The carbonyl content determined in PM muscle of standard turkeys was higher than that measured in the PM muscle of breeder turkeys. However, the difference between the two physiological stages was low. The sex had no effect on the carbonyl content in PM muscle. In the Sartorius (SART) muscle, the carbonyl content was a bit higher than that measured in the PM muscle. In SART muscle, the physiological stage had no effect. The females had a higher carbonyl content in SART muscle than the males, but the difference was low. The physiological stage had no effect on the thiol content in PM and SART muscles. The sex had no effect on the thiol content in PM muscle. On the other hand, the females had lower thiol content in SART muscle than males, suggesting a higher level of protein oxidation, as the ability to release thiol was affected, and confirming the results obtained with carbonyl. The TBARS value was higher in SART muscle compared to PM muscle, and corroborating the contents in lipids and haemininc pigments. It was also higher in breeder turkeys compared to standard turkeys. The sex had no effect on the lipid peroxidation in PM and SART muscles. 

### 3.3. Nutritional Quality of Meat (Table 3, Table 4 and Table 5)

The breeder turkeys had a higher lipid content in PM and IT muscle than standard animals (Table 3). The male breeders had a lower protein content in PM muscle than the other groups and the lowest protein content in IT muscle. The physiological stage had no effect on the iron and haeminic pigment (myoglobin and hemoglobin) contents in PM muscle whereas in IT muscle, the iron and haeminic pigment contents were higher in breeder turkeys compared to standard turkeys. The males had higher iron and haeminic pigment contents in PM and IT muscles than females. The iron and haeminic pigment contents in IT muscle were 2- to 3-fold higher to that measured in PM muscle. The coefficient of correlation between the haeminic pigment content in IT muscle and redness was 0.73 (*p* < 0.05).

The sex had no effect on the triglyceride, cholesterol, and phospholipid contents of PM muscle (Table 3). The physiological stage had no effect on the cholesterol content of PM muscle. By contrast, the breeder turkeys had higher triglyceride and phospholipid contents in PM muscle than standard turkeys. The sex had no effect on the cholesterol and phospholipid contents of IT muscle. The males had a higher triglyceride content in IT muscle than the females. The physiological stage had no effect on the phospholipid content in IT muscle. The breeder turkeys had a higher triglyceride content and a lower cholesterol content than the standard turkeys. 

The PM muscle had a high *n*-6 fatty acid (FA) content and the ratio *n*-6/*n*-3 FA was around 10–12 (Table 4). The sex had few effects on the FA composition of PM muscle. However, the breeder females had a higher saturated FA (SFA) content than breeder males. The breeder turkeys had a higher mon-unsaturated FA (MUFA) content and lower SFA and poly-unsaturated FA (PUFA) contents. 

The IT muscle had a fatty acid composition close to that described for PM muscle (Table 5). The ratio *n*-6/*n*-3 FA varied between 11 and 15. The sex had more effect on the FA composition of IT muscle, but the differences observed between males and females for one given physiological stage were low. The males had a content in C18:3 *n*-3 higher to that of females. The effect of physiological stage was significant for all FA except C20:5 *n*-3 and C22:4 *n*-6. The IT muscle of breeder turkeys had a higher MUFA content and lower SFA and PUFA contents to that of standard turkeys. 

### 3.4. Sensorial Quality of Meat (Table 6)

The thighs of breeder turkeys were judged more colored, less soft, less juicy, stringier, and more compact than those of standard animals. They were also less appreciated. There was no effect of the physiological stage on the oily sensation of cooked thighs nor on the global and rancid flavors, whose scores were very low. The thighs of males were judged more colored, juicier, and stringier than those of females. 

The breasts of breeder turkeys were judged less tender, less juicy, stringier, and less sticky to those of standard turkeys. They also had a flavor less acid and they were less appreciated, particularly those of male breeders. There was no effect of physiological stage on the color of cooked breast and global and rancid flavors, whose scores were very low. The breasts of males, particularly those of breeders, were less tender, stringier, and less sticky than the breasts of females. Their global flavor was lower, and they were less appreciated than those of females. 

The coefficients of correlation between the tenderness and stringiness scores and the shear-force value were −0.84 and 0.73, respectively (*p* < 0.05). 

### 3.5. Histological Characteristics of Pectoralis Major Muscle (Table 7, Figure 1 and Figure 2)

The females, particularly the standard ones, had lower CSA of muscle fibers and higher relative area occupied by collagen than males. The breeder turkeys, particularly the males, had a higher CSA of muscle fibers and lower relative area occupied by collagen than standard turkeys. The breeder females and the standard males had a comparable average CSA. The CSA of muscle fibers was correlated (*p* < 0.05) with the weight (0.87), the shear-force value (0.74), and the tenderness (−0.83) and stringiness (0.73) scores of PM muscle. The relative area occupied by collagen was correlated (*p* < 0.05) with the weight (−0.40), the shear-force value (−0.34), and CSA of muscle fibers (−0.42) of PM muscle. 

## 4. Discussion

### 4.1. Sex Effect

The turkey is characterized by a strong sexual dimorphism on body weight resulting in a higher weight of carcass and cut pieces for males compared to females. It was the same for the yields expressed relative to body weight. The coefficient multiplier between the breast weight of male breeders and that of female breeders was 3.48. For the CSA of muscle fibers of PM muscle, this coefficient was 2.02. For the standard turkeys, these coefficients were 2.43 and 1.79, respectively. This means that the breast weight difference between males and females was partially due to the hypertrophy of the muscle fibers but also due to a more important number and/or length of muscle fibers.

Concerning the technological quality, males slaughtered at older ages presented a lower pHu in PM and IT muscles than that measured for females, suggesting higher glycogen reserves [41]. The PM and IT muscles of males had higher haeminic pigment content and they were redder than those of females. The males had PM muscles displaying higher drip loss during a storage at 4 °C and higher cooking loss, resulting in a lower technological yield than that of females [41]. On the other hand, the protein oxidation in SART muscle during a storage at 4 °C was lower for males compared to females. 

### 4.2. Effect of Physiological Stage

The breeder turkeys slaughtered at older ages had higher body weight and weight of cut pieces than those of standard turkeys. Their carcass and breast yields were also higher, whereas the yield of thigh with drumstick was lower than those of standard turkeys. A study concerning the meat valuation of hens at the end of their laying cycle had shown rather the opposite, with a carcass yield relative to body weight of 60% and a breast yield of 11% [42]. The PM and IT muscles of breeder turkeys were redder and the content in haeminic pigment of IT muscle was higher than those of standard turkeys. During the sensory analysis, the thighs of breeder turkeys were also judged more colored. The thigh and breast muscles of hens and cocks slaughtered at 64 weeks of age were also redder compared to those of male and female chickens slaughtered at 6 weeks of age [43]. The same observation was reported for a comparison between breeder ducks slaughtered at 500 days of age and growing ducks slaughtered at 38 days of age [44]. The breast muscles of breeder turkeys were less tender than those of standard turkeys. They were also judged stringier. These differences were mainly explained by the difference in CSA of muscle fibers. The breast and thigh muscles of breeder ducks were also judged less tender than those of growing ducks [44]. The PM muscle of breeder males also had a lower protein content and a lower technological yield after the cured and cooked process. During the sensorial analysis, the thighs of breeder turkeys were judged less juicy than those of standard turkeys. The PM and IT muscles of breeder turkeys had higher lipid, triglyceride, and MUFA contents than those of standard turkeys. However, this had no effect on the flavor of cooked thighs evaluated during the sensorial analysis. On the reverse, the breeder males had the lowest flavor score. A lower meat tenderness and a higher carcass and intramuscular fatness were recorded in laying hens slaughtered at 56 weeks of age compared to broiler chickens [45]. The breast and thigh muscles of breeder ducks had higher protein, lipid, and MUFA contents than those of growing ducks [44]. They also had a higher flavor score. The breast muscles of breeders 64 weeks old were less juicy, less tender, and less tasty than those of chickens 6 weeks old [43]. The flavor score was also lower. 

## 5. Conclusions

The differences observed between males and females on one hand and between standard and breeder turkeys on the other hand mainly resulted from the difference in slaughter ages and sexual dimorphism on body weight. The meat of female breeders had characteristics close to those of standard turkeys, whereas the meat of male breeders was clearly distinguishable, particularly by displaying lower tenderness and water holding capacity. For the latter, the transformation and/or cooking processes must be clearly adapted by suggesting, for example. a use in minced meat to make loaf, or in cut pieces associated with a long cooking. The storage at −20 °C of breast and thigh muscles vacuum-packed for 5 to 7 months did not induce oxidation phenomena, which was confirmed by the oxidation measures of proteins and lipids.

## Figures and Tables

**Figure 1:**
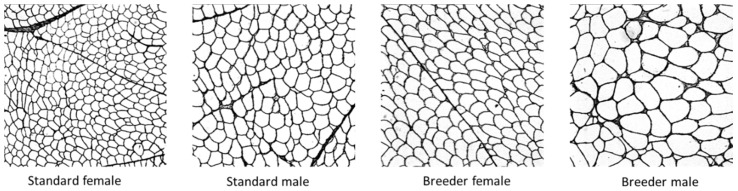
Cross-sectional area of Pectoralis major muscle from males and females of breeder and standard turkeys (*n* = 13, magnification ×5).

**Figure 2:**
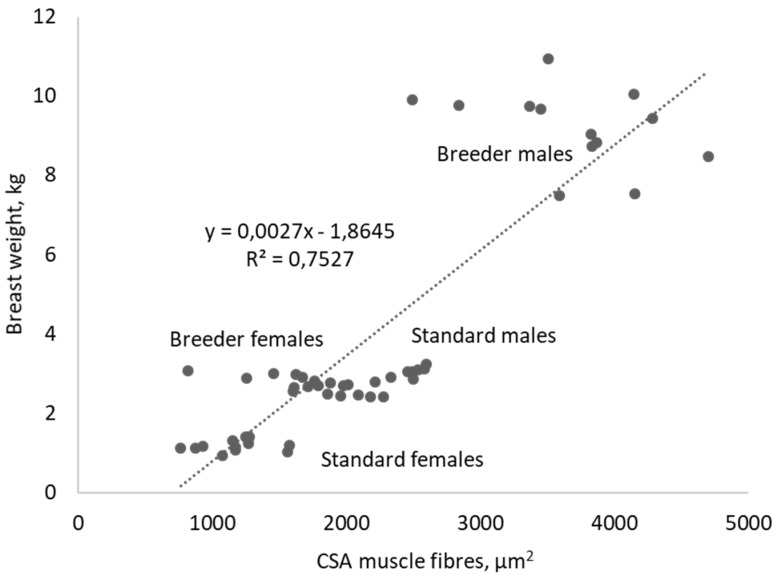
Relation between CSA of muscle fibers and breast weight from males and females of breeder and standard turkeys (*n* = 13).

**Table 1:** Meat yields of males and females of breeder and standard turkeys (*n* = 20).

	Female Breeders	Male Breeders	Standard Females	Standard Males	Physiological Stage Effect	Sex Effect	Interaction Effect
Slaughter age (days)	397	410	75	103			
Body weight at slaughter (kg)	10.42 ± 0.75 ^c^	32.67 ± 1.51 ^a^	5.89 ± 0.35 ^d^	13.48 ± 0.56 ^b^	0.001	0.001	0.001
Carcass weight (kg)	7.46 ± 0.56 ^c^	25.08 ± 1.33 ^a^	4.15 ± 0.31 ^d^	9.74 ± 0.42 ^b^	0.001	0.001	0.001
Breast weight (kg)	2.64 ± 0.23 ^c^	9.20 ± 0.99 ^a^	1.22 ± 0.13 ^d^	2.97 ± 0.19 ^b^	0.001	0.001	0.001
Thigh + drumstick weight (kg)	2.22 ± 0.18 ^c^	7.83 ± 0.78 ^a^	1.43 ± 0.10 ^d^	3.40 ± 0.19 ^b^	0.001	0.001	0.001
Carcass yield (%)	71.62 ± 1.09 ^c^	76.75 ± 1.58 ^a^	70.36 ± 1.59 ^d^	72.25 ± 1.36 ^b^	0.001	0.001	0.001
Breast yield (%)	25.31 ± 1.16 ^b^	28.15 ± 2.51 ^a^	20.69 ± 1.27 ^d^	22.01 ± 1.02 ^c^	0.001	0.001	0.001
Thigh + drumstick yield (%)	21.26 ± 0.70 ^b^	23.98 ± 2.26 ^a^	24.32 ± 0.73 ^a^	25.19 ± 0.91 ^a^	0.001	0.001	0.002

Yields are expressed as percentage of body weight at slaughter. The breasts include the two pectoral muscles (P. major and P. minor) without skin. The thighs and drumsticks include the bones and skin. ^a, b, c, d^ within a row, significant differences between groups with *p* < 0.05.

**Table 2:** Technological quality of meat from males and females of breeder and standard turkeys (*n* = 20).

	Female Breeders	Male Breeders	Standard Females	Standard Males	Physiological Stage Effect	Sex Effect	Interaction Effect
pHu PM	5.68 ± 0.08 ^b^	5.70 ± 0.13 ^ab^	5.77 ± 0.07 ^a^	5.68 ± 0.05 ^b^	0.09	0.04	0.005
pHu IT	6.03 ± 0.11	5.79 ± 0.12	6.14 ± 0.14	5.92 ± 0.10	0.001	0.001	0.66
L* PM	49.14 ± 3.01 ^a^	51.00 ± 3.24 ^a^	49.30 ± 2.07 ^a^	46.00 ± 2.21 ^b^	0.001	0.23	0.001
a* PM	−0.38 ± 0.90	0.48 ± 1.02	−0.75 ± 0.73	−0.62 ± 0.86	0.001	0.01	0.07
b* PM	7.69 ± 1.44 ^a^	6.27 ± 1.56 ^b^	7.06 ± 1.23 ^ab^	7.00 ± 1.31 ^ab^	0.87	0.02	0.03
L* IT	36.90 ± 3.50 ^b^	42.64 ± 4.48 ^a^	42.85 ± 2.03 ^a^	42.46 ± 2.02 ^a^	0.001	0.001	0.001
a* IT	5.63 ± 1.04 ^a^	5.97 ± 1.02 ^a^	2.08 ± 0.99 ^c^	4.03 ± 1.21 ^b^	0.001	0.001	0.001
b* IT	4.00 ± 1.00	2.57 ± 1.74	4.66 ± 1.09	3.85 ± 0.80	0.001	0.001	0.26
PM texture (N/cm^2^)	16.62 ± 1.65 ^b^	27.00 ± 4.12 ^a^	15.73 ± 1.63 ^b^	16.68 ± 1.78 ^b^	0.001	0.001	0.001
PM drip loss (%)	0.77 ± 0.34	1.29 ± 0.72	0.76 ± 0.31	0.97 ± 0.32	0.12	0.001	0.14
PM cooking loss (%)	11.08 ± 1.48 ^b^	15.29 ± 2.43 ^a^	8.59 ± 0.94 ^c^	9.78 ± 1.08 ^bc^	0.001	0.001	0.001
PM technological yield (%)	86.38 ± 2.16 ^a^	72.38 ± 4.68 ^c^	84.28 ± 2.72 ^ab^	81.35 ± 2.03 ^b^	0.001	0.001	0.001
PM carbonyl content	2.03 ± 0.38	2.14 ± 0.39	2.33 ± 0.30	2.26 ± 0.37	0.01	0.81	0.27
SART carbonyl content	3.43 ± 0.52	3.00 ± 0.42	3.21 ± 0.73	3.06 ± 0.56	0.52	0.03	0.27
PM thiol content	31.36 ± 6.96	35.76 ± 8.97	34.64 ± 6.84	35.99 ± 9.50	0.34	0.12	0.41
SART thiols content	34.84 ± 4.02	37.64 ± 4.81	37.03 ± 3.59	37.82 ± 4.40	0.21	0.06	0.29
PM TBARS value	0.80 ± 0.35	0.79 ± 0.25	0.44 ± 0.19	0.66 ± 0.34	0.001	0.13	0.09
SART TBARS value	1.34 ± 0.48	1.30 ± 0.45	0.89 ± 0.31	1.06 ± 0.38	0.001	0.48	0.26

PM = Pectoralis major; IT = Iliotibialis superficialis; SART = Sartorius; L* = lightness; a* = redness; b* = yellowness; PM texture was estimated by measuring the shear-force value (N/cm^2^). The drip loss was measured after 6 days storage of breast cutlets at 4 °C. The technological yield was estimated on cured–cooked samples of breast muscle. The carbonyl content was expressed as nmol DNPH (2,4-dinitrophenylhydrazine) incorporated/mg protein. The thiol content was expressed as nmol/mg protein. The TBARS (Thio-Barbituric Acid Reactive Substances) value was expressed as mg equivalent MDA/g muscle. MDA = malondialdehyde; ^a, b, c^ within a row, significant differences between groups with *p* < 0.05.

**Table 3:** Nutritional quality of meat from males and females of breeder and standard turkeys (*n* = 10).

	Female Breeders	Male Breeders	Standard Females	Standard Males	Physiological Stage Effect	Sex Effect	Interaction Effect
Dry matter PM (%)	27.70 ± 0.87	24.11 ± 1.82	25.60 ± 0.70	26.16 ± 0.51	0.001	0.26	0.23
Proteins PM (%)	24.72 ± 0.87 ^a^	21.68 ± 1.80 ^b^	25.21 ± 0.89 ^a^	25.41 ± 0.52 ^a^	0.001	0.001	0.001
Lipides PM (%)	3.01 ± 0.86	2.51 ± 0.96	1.01 ± 0.33	1.02 ± 0.15	0.001	0.26	0.23
Ashes PM (%)	1.08 ± 0.04 ^b^	1.05 ± 0.04 ^b^	1.42 ± 0.05 ^a^	1.10 ± 0.13 b	0.001	0.001	0.001
Iron (µg/g PM)	3.03 ± 0.56	3.42 ± 1.31	2.37 ± 1.15	3.77 ± 1.45	0.65	0.01	0.14
Myoglobin (µg/g PM)	922 ± 170	1042 ± 399	722 ± 349	1147 ± 441	0.65	0.01	0.14
Dry matter IT (%)	26.14 ± 0.46 ^a^	24.92 ± 1.05 ^ab^	23.69 ± 1.12 ^b^	24.52 ± 0.75 ^ab^	0.001	0.50	0.001
Proteins IT (%)	22.70 ± 0.31 ^a^	20.25 ± 1.03 ^b^	21.79 ± 0.67 ^ab^	21.39 ± 0.95 ^b^	0.65	0.001	0.001
Lipids IT (%)	3.50 ± 0.69	4.20 ± 1.13	2.56 ± 0.73	2.91 ± 0.77	0.001	0.06	0.52
Ash IT (%)	1.12 ± 0.04 ^a^	1.08 ± 0.05 ^ab^	1.02 ± 0.07 ^b^	1.05 ± 0.04 ^b^	0.001	0.57	0.02
Iron (µg/g IT)	10.33 ± 2.48	12.70 ± 2.51	5.58 ± 1.68	8.21 ± 2.35	0.001	0.001	0.84
Myoglobin (µg/g IT)	3143 ± 756	3865 ± 765	1699 ± 511	2500 ± 714	0.001	0.001	0.84
Triglycerides PM (%)	2.51 ± 0.81	2.02 ± 0.90	0.54 ± 0.21	0.64 ± 0.13	0.001	0.33	0.14
Cholesterol PM (%)	0.04 ± 0.02 ^b^	0.07 ± 0.02 ^a^	0.05 ± 0.02 ^ab^	0.04 ± 0.01 ^b^	0.31	0.15	0.001
Phospholipids PM (%)	0.46 ± 0.08	0.42 ± 0.07	0.40 ± 0.13	0.34 ± 0.07	0.01	0.09	0.77
Triglycerides IT (%)	2.76 ± 0.57	3.29 ± 0.95	1.77 ± 0.59	2.16 ± 0.70	0.001	0.05	0.77
Cholesterol IT (%)	0.07 ± 0.03 ^b^	0.09 ± 0.04 ^ab^	0.14 ± 0.04 ^a^	0.08 ± 0.03 ^b^	0.01	0.10	0.001
Phospholipids IT (%)	0.67 ± 0.12	0.82 ± 0.25	0.65 ± 0.11	0.67 ± 0.35	0.24	0.25	0.38

PM and IT = Pectoralis major and Iliotibialis superficialis muscles, respectively; ^a, b^ within a row, significant differences between groups with *p* < 0.05.

**Table 4:** Fatty acid (FA) composition of P. major muscle from males and females of breeder and standard turkeys (% total FA; *n* = 10).

	Female Breeders	Male Breeders	Standard Females	Standard Males	Physiological Stage Effect	Sex Effect	Interaction Effect
C14:0	0.90 ± 0.54 ^a^	0.54 ± 0.06 ^ab^	0.41 ± 0.06 ^b^	0.52 ± 0.08 ^ab^	0.006	0.18	0.01
C14:1	0.10 ± 0.04 ^ab^	0.14 ± 0.06 ^a^	0.09 ± 0.06 ^ab^	0.06 ± 0.02 ^b^	0.004	0.74	0.05
C16:0	26.11 ± 0.83 ^a^	22.05 ± 1.15 ^c^	24.03 ± 1.03 ^bc^	25.60 ± 0.53 ^ab^	0.001	0.18	0.03
C16:1	3.93 ± 0.79 ^a^	4.12 ± 0.76 ^a^	2.07 ± 0.68 ^b^	1.28 ± 0.46 ^b^	0.001	0.18	0.03
C18:0	7.09 ± 0.54	7.11 ± 1.26	11.21 ± 1.49	9.81 ± 1.46	0.001	0.09	0.08
C18:1	35.63 ± 1.65	34.94 ± 1.62	24.88 ± 1.77	26.06 ± 0.97	0.001	0.61	0.06
C18:2 *n*-6	22.02 ± 1.23 ^c^	25.89 ± 2.20 ^bc^	29.38 ± 1.77 ^ab^	29.68 ± 1.43 ^a^	0.001	0.001	0.01
C18:3 *n*-3	1.17 ± 0.15	1.95 ± 0.40	1.77 ± 0.36	2.15 ± 0.40	0.001	0.001	0.07
C20:0	0.05 ± 0.03	0.07 ± 0.02	0.09 ± 0.02	0.11 ± 0.02	0.001	0.10	0.14
C20:1	0.20 ± 0.06	0.25 ± 0.06	0.17 ± 0.02	0.17 ± 0.02	0.001	0.10	0.14
C20:4 *n*-6	1.53 ± 0.46 ^c^	1.92 ± 1.20 ^bc^	4.53 ± 1.05 ^a^	3.42 ± 0.91 ^ab^	0.001	0.24	0.02
C20:5 *n*-3	0.62 ± 0.59	0.48 ± 0.74	0.10 ± 0.07	0.09 ± 0.04	0.01	0.60	0.66
C22:4 *n*-6	0.11 + 0.03	0.10 + 0.07	0.22 + 0.05	0.20 + 0.04	0.001	0.37	0.52
C22:5 *n*-3	0.12 ± 0.03 ^c^	0.28 ± 0.15 ^bc^	0.69 ± 0.19 ^a^	0.57 ± 0.13 ^ab^	0.001	0.62	0.01
C22:6 *n*-3	0.41 ± 0.17	0.16 ± 0.15	0.36 ± 0.11	0.27 ± 0.08	0.49	0.001	0.07
SFA	34.16 ± 0.82 ^a^	29.77 ± 1.79 ^b^	35.74 ± 2.16 ^a^	36.04 ± 1.55 ^a^	0.001	0.001	0.001
MUFA	39.86 ± 1.43	39.45 ± 2.10	27.20 ± 2.21	27.57 ± 1.38	0.001	0.96	0.50
PUFA	25.99 ± 1.05 ^c^	30.78 ± 2.55 ^b^	37.05 ± 2.63 ^a^	36.38 ± 1.28 ^a^	0.001	0.01	0.001
*n*-6 FA	23.66 ± 0.98 ^c^	27.91 ± 2.18 ^b^	34.13 ± 2.31 ^a^	33.31 ± 1.11 ^a^	0.001	0.01	0.001
*n*-3 FA	2.32 ± 0.57	2.87 ± 0.89	2.92 ± 0.36	3.08 ± 0.24	0.03	0.06	0.29
*n*-6 FA/*n*-3 FA	10.70 ± 2.47	10.27 ± 2.14	11.79 ± 0.91	10.87 ± 0.68	0.13	0.23	0.65

SFA, MUFA, PUFA = Saturated, mono-unsaturated, and poly-unsaturated fatty acids, respectively; ^a, b, c^ within a row, significant differences between groups with *p* < 0.05.

**Table 5:** Fatty acid (FA) composition of I. superficialis muscle from males and females of breeder and standard turkeys (% total FA, *n* = 10).

	Female Breeders	Male Breeders	Standard Females	Standard Males	Physiological Stage Effect	Sex Effect	Interaction Effect
C14:0	0.68 ± 0.04 ^a^	0.54 ± 0.08 ^b^	0.52 ± 0.04 ^b^	0.60 ± 0.04 ^a^	0.01	0.07	0.001
C14:1	0.12 ± 0.03	0.13 ± 0.02	0.07 ± 0.02	0.05 ± 0.02	0.001	0.97	0.14
C16:0	24.60 ± 0.49 ^ab^	21.32 ± 1.05 ^c^	23.32 ± 1.15 ^bc^	25.13 ± 0.62 ^a^	0.001	0.01	0.001
C16:1	3.44 ± 0.57 ^ab^	3.89 ± 0.77 ^a^	2.28 ± 0.78 ^bc^	1.41 ± 0.48 ^c^	0.001	0.33	0.01
C18:0	8.39 ± 0.51	7.95 ± 0.84	9.64 ± 0.93	8.89 ± 0.93	0.001	0.03	0.57
C18:1	32.52 ± 0.73	33.26 ± 1.24	24.69 ± 2.03	25.87 ± 0.84	0.001	0.03	0.60
C18:2 *n*-6	25.51 ± 1.06 ^b^	27.91 ± 1.52 ^b^	32.55 ± 2.17 ^a^	32.10 ± 0.72 ^a^	0.001	0.001	0.01
C18:3 *n*-3	1.17 ± 0.13 ^c^	1.85 ± 0.23 ^b^	2.43 ± 0.27 ^a^	2.67 ± 0.19 ^a^	0.001	0.001	0.01
C20:0	0.08 ± 0.01	0.08 ± 0.01	0.10 ± 0.02	0.11 ± 0.02	0.001	0.21	0.10
C20:1	0.22 ± 0.03 ^ab^	0.27 ± 0.04 ^a^	0.18 ± 0.02 ^b^	0.19 ± 0.02 ^b^	0.001	0.001	0.01
C20:4 *n*-6	2.37 ± 0.29 ^ab^	2.33 ± 0.70 ^b^	3.35 ± 0.95 ^a^	2.32 ± 0.56 ^b^	0.03	0.02	0.03
C20:5 *n*-3	0.07 ± 0.06	0.06 ± 0.04	0.08 ± 0.09	0.10 ± 0.04	0.15	0.98	0.49
C22:4 *n*-6	0.19 + 0.04 ^a^	0.11 + 0.03 ^b^	0.15 + 0.03 ^ab^	0.12 + 0.04 ^b^	0.35	0.001	0.04
C22:5 *n*-3	0.12 ± 0.02 ^c^	0.21 ± 0.06 ^b^	0.45 ± 0.13 ^a^	0.32 ± 0.08 ^ab^	0.001	0.52	0.001
C22:6 *n*-3	0.54 ± 0.14 ^a^	0.12 ± 0.04 ^c^	0.21 ± 0.08 ^b^	0.12 ± 0.05 ^c^	0.001	0.001	0.001
SFA	33.75 ± 0.94 ^a^	29.88 ± 1.16 ^b^	33.58 ± 1.19 ^a^	34.74 ± 0.82 ^a^	0.001	0.001	0.001
MUFA	36.29 ± 0.97 ^a^	37.55 ± 1.85 ^a^	27.21 ± 2.75 ^b^	27.52 ± 1.29 ^b^	0.001	0.38	0.01
PUFA	29.89 ± 0.97 ^b^	32.51 ± 1.93 ^b^	39.13 ± 3.20 ^a^	37.65 ± 1.08 ^a^	0.001	0.38	0.01
*n*-6 FA	28.07 ± 0.94 ^b^	30.34 ± 1.93 ^b^	36.05 ± 2.85 ^a^	34.54 ± 1.03 ^a^	0.001	0.02	0.03
*n*-3 FA	1.83 ± 0.12 ^c^	2.18 ± 0.19 ^b^	3.09 ± 0.38 ^a^	3.10 ± 0.11 ^a^	0.001	0.02	0.03
*n*-6 FA/*n*-3 FA	15.41 ± 0.98	14.05 ± 1.65	11.76 ± 0.84	11.14 ± 0.42	0.001	0.01	0.28

SFA, MUFA, PUFA = saturated, mono-unsaturated, and poly-unsaturated fatty acids, respectively; ^a, b, c^ within a row, significant differences between groups with *p* < 0.05.

**Table 6:** Sensorial analysis of cooked thigh and breast meat from males and females of breeder and standard turkeys (*n* = 10).

	Female Breeders	Male Breeders	Standard Females	Standard Males	Physiological Stage Effect	Sex Effect	Interaction Effect
Thighs							
Colour	4.78 ± 1.41 ^b^	6.29 ± 1.49 ^a^	3.04 ± 1.32 ^c^	3.43 ± 1.43 ^c^	0.001	0.001	0.001
Tenderness	2.45 ± 1.03 ^c^	2.99 ± 1.45 ^b^	4.38 ± 1.34 ^a^	4.01 ± 1.21 ^a^	0.001	0.46	0.001
Juiciness	1.54 ± 0.85	1.81 ± 1.21	2.22 ± 0.97	2.36 ± 1.32	0.001	0.05	0.56
Stinginess	2.18 ± 1.15	2.86 ± 1.40	1.47 ± 1.18	1.99 ± 1.21	0.001	0.001	0.47
Compactness	3.03 ± 1.75	2.60 ± 1.97	1.64 ± 1.11	1.76 ± 1.25	0.001	0.29	0.07
Oily sensation	1.35 ± 1.19	1.50 ± 1.42	1.54 ± 1.29	1.47 ± 1.14	0.51	0.73	0.37
Global flavour	3.92 ± 1.05	4.08 ± 1.01	3.83 ± 1.00	3.88 ± 0.95	0.13	0.29	0.55
Rancid flavour	0.37 ± 0.41	0.37 ± 0.37	0.36 ± 0.39	0.32 ± 0.29	0.36	0.45	0.63
Global appreciation	2.52 ± 1.26	2.51 ± 1.21	3.38 ± 1.54	3.58 ± 1.33	0.001	0.46	0.39
Breast							
Colour	2.25 ± 1.43	2.49 ± 1.35	2.23 ± 1.37	2.45 ± 1.36	0.83	0.07	0.93
Tenderness	3.79 ± 1.16 ^b^	2.07 ± 1.14 ^c^	4.71 ± 1.39 ^a^	4.41 ± 1.33 ^a^	0.001	0.001	0.001
Juiciness	3.05 ± 1.46	2.78 ± 1.61	3.33 ± 1.50	3.15 ± 1.31	0.02	0.11	0.74
Stringiness	2.28 ± 1.26 ^b^	3.15 ± 1.61 ^a^	1.69 ± 1.24 ^c^	2.03 ± 1.21 ^bc^	0.001	0.001	0.04
Sticky	1.63 ± 1.13	1.11 ± 1.00	1.83 ± 1.33	1.71 ± 1.19	0.001	0.004	0.07
Global flavour	3.41 ± 0.94 ^a^	3.00 ± 1.04 ^b^	3.37 ± 1.03 ^a^	3.38 ± 0.99 ^a^	0.06	0.03	0.03
Acid flavour	1.11 ± 0.80	1.03 ± 1.00	1.36 ± 1.13	1.26 ± 1.02	0.01	0.31	0.93
Rancid flavour	0.33 ± 0.29	0.37 ± 0.42	0.33 ± 0.34	0.29 ± 0.28	0.23	0.82	0.23
Global appréciation	2.27 ± 1.16 ^a^	1.60 ± 1.03 ^b^	2.28 ± 1.23 ^a^	2.42 ± 1.25 ^a^	0.001	0.02	0.001

^a,b,c^ within a row, significant differences between groups with *p* < 0.05.

**Table 7:** Relative area occupied by collagen and cross-sectional area (CSA) of muscle fibers of Pectoralis major muscle from males and females of breeder and standard turkeys (*n* = 13).

	Female Breeders	Male Breeders	Standard Females	Standard Males	Physiological Stage Effect	Sex Effect	Interaction Effect
Collagen, %	19.09 ± 3.20	17.06 ± 2.37	20.75 ± 1.88	18.85 ± 3.50	0.03	0.02	0.94
AST, µm^2^	1826 ± 419 ^b^	3695 ± 593 ^a^	1181 ± 238 ^c^	2117 ± 439 ^b^	0.001	0.001	0.001

^a, b, c^ within a row, significant differences between groups with *p* < 0.05.
